# N-acetylcysteine alleviates cadmium-induced placental endoplasmic reticulum stress and fetal growth restriction in mice

**DOI:** 10.1371/journal.pone.0191667

**Published:** 2018-01-26

**Authors:** Min-Yin Guo, Hua Wang, Yuan-Hua Chen, Mi-Zhen Xia, Cheng Zhang, De-Xiang Xu

**Affiliations:** 1 Department of Toxicology, School of Public Health, Anhui Medical University, Hefei, China; 2 Department of Histology and Embryology, Anhui Medical University, Hefei, China; 3 Life Science College, Anhui Medical University, Hefei, China; University of Kentucky, UNITED STATES

## Abstract

Cadmium (Cd) is a developmental toxicant that induces fetal growth restriction (FGR). Placental endoplasmic reticulum (ER) stress is associated with FGR. This study investigated the effects of N-acetylcysteine (NAC) on Cd-induced placental ER stress and FGR. Pregnant mice were intraperitoneally injected with CdCl_2_ daily from gestational day (GD)13 to GD17. As expected, Cd reduced fetal weight and crown-rump length. Cd decreased the internal space of blood vessels in the placental labyrinth layer and inhibited placental cell proliferation. Several genes of growth factors, such as *Vegf-a*, *placental growth factor*, *Igf1* and *Igf1r*, and several genes of nutrient transport pumps, such as *Glut1*, *Fatp1* and *Snat2*, were down-regulated in placenta of Cd-treated mice. Moreover, Cd evoked placental ER stress. Of interest, NAC alleviated Cd-induced FGR. Additional experiment showed that NAC inhibited Cd-induced impairment of placental development and placental ER stress. Therefore, NAC may be exploited for prevention of Cd-induced placental insufficiency and FGR.

## Instruction

Cadmium (Cd) is a kind of toxic heavy metals. Cd contamination is pervasive in soil and food, which has been widely concerned as one of major environmental problem. Increasing cohort studies demonstrated that high Cd level during pregnancy is associated with increased risks of preterm delivery, low birth weight and fetal growth restriction [1–4]. Several animal experiments also found that Cd exposure at middle gestational stage resulted in neural tube defects in mice [5–9]. Moreover, Cd exposure at late gestational stage resulted in fetal growth restriction in mice [9–12]. Nevertheless, the mechanism for Cd induced developmental toxicity remains unclear.

The placenta is important for sustaining the growth of the fetuses. Increasing evidence has demonstrated that the defects in placental development result in fetal death, birth defects and fetal growth restriction [13]. The endoplasmic reticulum (ER) is an important organelle that is required for cell survival and normal cellular function [14]. The ER is sensitive to alterations in cellular homeostasis. Several recent studies demonstrated that Cd was an inducer of ER stress [15,16]. On the other hand, an increased ER stress was associated with the impaired placental development and fetal growth restriction [17–22]. Our recent report showed that reactive oxygen species (ROS)-mediated placental ER stress was associated with Cd-induced teratogenicity in mice [8].

N-acetylcysteine (NAC) is a direct antioxidant and a GSH precursor [23]. Previous study showed that NAC prevented Cd-induced oxidative stress [24]. The aim of the present study was to investigate whether NAC protects against Cd-induced placental ER stress and fetal growth restriction in mice. Our results found that NAC attenuated Cd-induced placental ER stress. Importantly, NAC alleviated Cd-induced placental development and fetal growth restriction.

## Materials and methods

### Chemicals and reagents

CdCl_2_ and NAC were from Sigma Chemical Co. (St. Louis, MO). Antibody against CD34 was from Abcam (Cambridge, MA). Antibodies against phosphor-eukaryotic translation initiation factor (eIF)2α, eIF2α and GRP78 were from Cell Signaling Technology (Beverley, MA). Antibody against β-actin was from Santa Cruz Biotechnologies (Santa Cruz, CA). Antibodies against phosphor-inositol requiring ER-to-nucleus signal kinase (IRE) 1α and IRE1α were from Pierce Biotechnology (Rockford, IL). Chemiluminescence (ECL) detection kit was from Pierce Biotechnology (Rockford, IL). TRI reagent was from Molecular Research Center, Inc (Cincinnati, Ohio). RNase-free DNase was from Promega Corporation (Madison, WI). All other reagents were purchased from Sigma Chemical Co. (St. Louis, MO) if not otherwise stated.

### Animals and treatments

The CD-1 mice (8~10 week-old) were purchased from Beijing Vital River whose foundation colonies were all introduced from Charles River Laboratories, Inc. Mice take food and water freely and were maintained on a 12-h dark/light cycle in a ambient temperature 23±3°C and humidity 50 ± 5%. Females and males were mated at a ratio of 2: 1. Females were checked in the following morning, and the presence of a vaginal plug was considered as gestational day (GD) 0. This study consisted of two experiments. Experiment 1. To investigate the effects of NAC on Cd-induced ER stress and FGR in mice, pregnant mice were divided randomly into four groups. In Cd group, pregnant mice were i.p. injected with CdCl_2_ (1.0 mg/kg) daily from GD13 to GD17. In NAC+Cd group, pregnant mice were i.p. injected with NAC (1.0 g/kg) daily from GD12 to GD17, one dose (300 mg/kg) at 8 h before CdCl_2_ injection, one (400 mg/kg) at 30 min before CdCl_2_ injection, another (300 mg/kg) at 8 h after CdCl_2_ injection. The saline-treated or NAC-treated pregnant mice served as controls. The doses of CdCl_2_ used in this study referred to others [6,7]. The doses of NAC used in this study referred to others [25]. All dams were anesthetized with pentobarbital (75 mg/kg, i.p.) treatments and sacrificed on GD18. The uterine horns were exposed and weighed. Live, dead and resorbed fetuses were counted. Live fetuses were sexed and weighed. The rate of FGR was calculated referred to others [26]. Placentas were weighed and then collected for measurement of histopathology, immunohistochemistry and ER stress. Experiment 2. To investigate the effects of NAC on Cd-induced down-regulation of growth factors and nutrient transport pumps in mouse placenta, twenty-four pregnant mice were divided randomly into four groups. In Cd group, pregnant mice were i.p. injected with CdCl_2_ (1.0 mg/kg) daily from GD13 to GD15. In NAC+Cd group, pregnant mice were injected with NAC (1.0 g/kg, i.g.) daily from GD12 to GD15, one dose (300 mg/kg) at 8 h before CdCl_2_ injection, one (400 mg/kg) at 30 min before CdCl_2_ injection, another (300 mg/kg) at 8 h after CdCl_2_ injection. The saline-treated or NAC-treated pregnant mice served as controls. All dams were anesthetized with pentobarbital (75 mg/kg, i.p.) treatments and sacrificed on GD15. Placentas were collected for real-time RT-PCR. This work was approved by the Association of Laboratory Animal Sciences and the Center for Laboratory Animal Sciences at Anhui Medical University (Permit Number: 15–0013). All procedures on mice followed the guidelines for humane treatment set by the Association of Laboratory Animal Sciences and the Center for Laboratory Animal Sciences at Anhui Medical University.

### Isolation of total RNA and real-time RT-PCR

Total RNA was extracted using TRI reagent and reverse-transcribed with AMV (Promega). Real-time RT-PCR was performed with a LightCycler 480 SYBR Green I kit (Roche Diagnostics GmbH) using gene-specific primers as listed in Table 1. The levels of mRNA were normalized using *18S*. The comparative C_T_ method was used to determine the amount of target, normalized to an internal reference (*18S*) and relative to a calibrator (2^−△△Ct^) using the Lightcycler480 software (Roche, version 1.5.0).

**Table 1:** Oligonucleotide sequences and size of primers.

Genes	Sequences	Sizes (bp)
***18S***	Forward: 5’- GTAACCCGTTGAACCCCATT-3’	151
	Reverse: 5’- CCATCCAATCGGTAGTAGCG-3’	
***Vegf-a***	Forward: 5’-TATTCAGCGGACTCACCAGC-3’	156
	Reverse: 5’-AACCAACCTCCTCAAACCGT-3’	
***Pgf***	Forward: 5’-ACTTGGGAACACAAGAAGCCT-3’	131
	Reverse: 5’-CGACCCCACACTTCGTTGAA-3’	
***Igf1***	Forward: 5’-AAGGCAGTTTACCCAGGCTC-3’	125
	Reverse: 5’-GGCCGAGGTGAACACAAAAC-3’	
***Igf2***	Forward: 5’-CTTCAGCAGCGTCCACTTCA-3’	105
	Reverse: 5’-TTGGTACCACAAGGCCGAAG-3’	
***Igf1r***	Forward: 5’-CCAAGCTCACCGTCATCACT-3’	110
	Reverse: 5’-GAAGAGTTTCCAGCCACGGA-3’	
***Glut1***	Forward: 5’-ACCATCTTGGAGCTGTTCCG-3’	131
	Reverse: 5’-GCCTTCTCGAAGATGCTCGT-3’	
***Fatp1***	Forward: 5’-CGCCGATGTGCTCTATGACT-3’	138
	Reverse: 5’-ACACAGTCATCCCAGAAGCG-3’	
***Fatp4***	Forward: 5’-GGCTCAGGGGCCAATAAACT-3’	102
	Reverse: 5’-TCCCAAGGGCTAAGCGAAAG-3’	
***Snat2***	Forward: 5’-ACCTCACCTGCTCGTCAAAG-3’	117
	Reverse: 5’-TGGTTGTCATGGCACCTCTC-3’	
***Snat4***	Forward: 5’- GGGCAGGGAATTAAGCTGGT-3’	156
	Reverse: 5’- ACCCTTCCTTCGCTACTTTGC-3’	
***Pcft***	Forward: 5’-CTACCCTACCTCACCAGCCT-3’	119
	Reverse: 5’-GCAAACGCAAAGACCACCAT-3’	
***Rfc-1***	Forward: 5’-TGGGTGTTGTAGTCTGCGTG-3’	114
	Reverse: 5’-CACTCCACCTTGCACTACCC-3’	

### Immunoblots

Fifty milligram placenta tissue was homogenized in 300 μl lysis buffer. Total lysate was separated electrophoretically by SDS-PAGE and transferred to a polyvinylidene fluoride membrane. The membranes were incubated for 2 h with the following antibodies: GRP78, peIF2α, eIF2α, pIRE1α and IRE1α. β-actin was used as a loading control. After washes in DPBS, the membranes were incubated for 2 h with goat anti-rabbit or goat anti-mouse IgG. The membranes were then washed for four times in DPBS, followed by signal development using an ECL detection kit.

### Immunohistochemistry

Placental sections were dewaxed and rehydrated. Antigen retrieval was performed by pressure cooking slides in 0.01 M citrate buffer (pH 6.0). After nonspecific binding sites were blocked with 5% normal bovine serum, slides were incubated for 12 h with polyclonal antibody Ki67 or CD34 at 4°C. After washing in TBS, slides were incubated with the biotin conjugated goat anti-rabbit IgG for 30 min, and then horseradish peroxidase-labeled avidin-biotin complex for 30 min. Immunostaining was developed by application of diaminobenzidine. Ki67-positive cells were counted in twelve randomly selected fields from each slide at a magnification of ×400.

### Placental glutathione content

Placental glutathione (GSH) content was determined by the method of Griffith [27].

### Statistical analysis

The litter was considered the unit for statistical comparison among different groups. Rates of fetal growth restriction were calculated per litter and then averaged per group. For fetal weight and crown-rump length, the means were calculated per litter and then averaged per group. All quantified data were expressed as means ± SEM. ANOVA and the Student-Newmann-Keuls post hoc test were used to determine differences among different groups. *P* < 0.05 was considered statistically significant.

## Results

### NAC attenuates Cd-induced fetal growth restriction

No pregnant mice were dead and no preterm delivery was observed in pregnant mice intraperitoneally injected with CdCl_2_. The effects of maternal Cd exposure on fetal outcomes are presented in Table 2. There was no significant difference on the number of resorptions per litter, live fetuses per litter and dead fetuses per litter among different groups. As expected, maternal Cd exposure at late gestational stage did not induce external malformations in fetuses. The effects of maternal Cd exposure at late gestational stage on fetal weight and crown-rump length are shown in Table 2. The fetal weight in the Cd group was lower as compared with controls. Correspondingly, crown-rump length was obviously reduced in the Cd group (Table 2). Further analysis showed that the rate of FGR per litter was markedly elevated in the Cd group (Table 2). The effects of NAC on Cd-induced fetal growth restriction were then analyzed. As shown in Table 2, NAC significantly attenuated Cd-induced reduction of fetal weight and crown-rump length. Correspondingly, NAC significantly attenuated Cd-induced elevation of FGR per litter.

**Table 2:** Fetal outcomes among different groups.

	Control	NAC	Cd	Cd+NAC
**Number of litters(n)**	19	14	17	20
**Resportions of per litter (n)**	0.4±0.2	0.3±0.2	0.5±0.2	0.5±0.2
**Death fetuses per litter (n)**	0.3±0.1	0.4±0.2	0.5±0.2	0.5±0.2
**Live fetuses per litter (n)**	12.7±0.5	13.0±0.2	13.1±0.6	12.4±0.4
**Fetal weight (g)**	1.397±0.023	1.371±0.026	1.247±0.017**	1.309±0.012^#^
**Crown-rump length (mm)**	24.46±0.16	24.35±0.24	22.92±0.17**	23.56±0.16^#^
**FGR per litter (%)**	10.5±2.7	14.6±4.5	51.9±8.2**	23.0±5.0^#^
**Placenta weight(g)**	0.100±0.003	0.095±0.003	0.091±0.003*	0.095±0.003

* *P*<0.05

**P<0.01 as compared with the control

^#^ *P*<0.05 as compared with Cd group.

### NAC alleviates Cd–induced impairment of placental development

**Fig 1:**
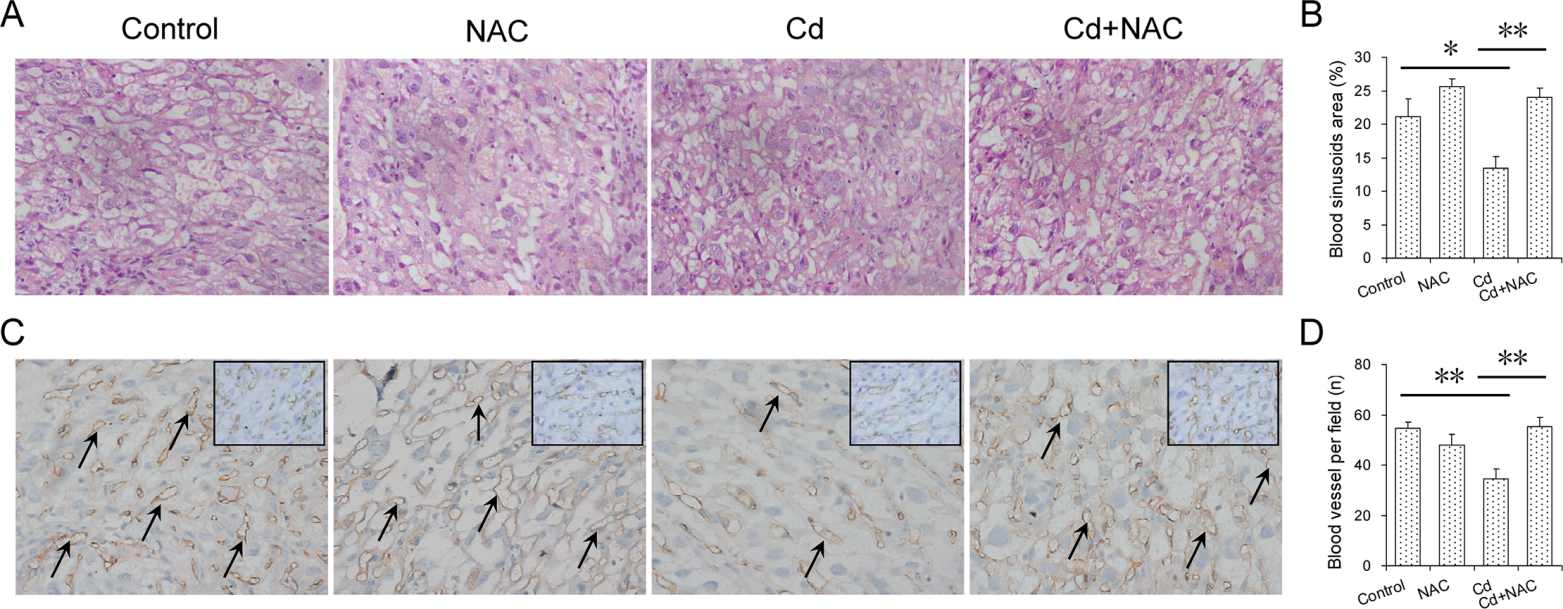
Effects of NAC on Cd-induced impairment of placental vascular space. All pregnant mice except controls were i.p. injected with CdCl_2_ (1.0 mg/kg) daily from GD13 to GD17. In NAC+Cd group, pregnant mice were i.p. injected with NAC (1.0 g/kg) daily from GD12 to GD17, one dose (300 mg/kg) at 8 h before CdCl_2_ injection, one (400 mg/kg) at 30 min before CdCl_2_ injection, another (300 mg/kg) at 8 h after CdCl_2_ injection. Placentas were collected on GD 18. (A) Placental cross sections were stained with H & E. Original magnification: 200×. (B) Vascular area in the labyrinthine layer was estimated from two nonconsecutive sections in each placenta using the public domain NIH Image J Program. The blood sinusoid area (%) was calculated as the ratio between the number of pixels covered by the area defined by the threshold and the overall number of pixels in the image. (C) Placental CD34 was analyzed using immunohistochemistry. Representative photomicrographs of histological specimens in the labyrinthine layer among different groups. Original magnification: 200×. Arrows indicate CD34-positive blood vessels. The images using black line were captured at magnification of 400×. (D) CD34-positive blood vessels were counted. (B and D) All data were expressed as means ± SEM. (N = 14~20). * *P*<0.05, **P<0.01.

**Fig 2:**
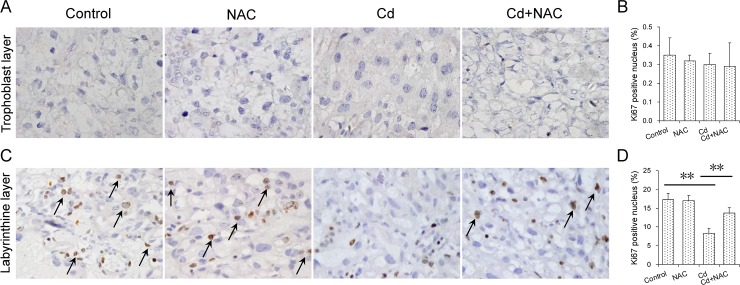
Effects of NAC on Cd-induced inhibition of placental cell proliferation. All pregnant mice except controls were i.p. injected with CdCl_2_ (1.0 mg/kg) daily from GD13 to GD17. In NAC+Cd group, pregnant mice were i.p. injected with NAC (1.0 g/kg) daily from GD12 to GD17, one dose (300 mg/kg) at 8 h before CdCl_2_ injection, one (400 mg/kg) at 30 min before CdCl_2_ injection, another (300 mg/kg) at 8 h after CdCl_2_ injection. Placentas were collected on GD 18. Placental cell proliferation was observed in Ki67 immunohistochemically stained placental sections. Sections were counterstained with hematoxylin. Arrows indicate Ki67-positive cells. (A) Placental cross section from trophoblast layer. (B) Percentage of Ki67-positive cells. (C) Placental cross section from labyrinthine layer. (D) Percentage of Ki67-positive cells. All data were expressed as means ± SEM. (N = 6). **P<0.01.

The effects of maternal Cd exposure at late gestational stage on placenta weight and histopathology were analyzed. As shown in Table 2, placenta weight was lower in Cd-exposed mice as compared with controls. Histopathology showed that the internal space of maternal and fetal blood vessels in the labyrinth layer was markedly reduced in the placenta of mice exposed to Cd (Fig 1A and 1B). Further analysis observed that the number of fetal blood vessels, as determined by CD34 immunostaining, was significantly reduced in the placenta of mice exposed to Cd (Fig 1C and 1D). The effects of NAC on Cd-induced impairment of placental vascular space were then analyzed. Of interest, NAC inhibited Cd-induced impairment of placental vascular space in the labyrinth layer (Fig 1). The effects of maternal Cd exposure at late gestational stage on cell proliferation in the placenta are presented in Fig 2. As expected, Ki67-positive cells in the trophoblast layer were reduced in the placenta of mice treated with Cd (Fig 2A and 2B). In addition, Ki67-positive cells in the labyrinthine layer were reduced in the placenta of mice treated with Cd (Fig 2C and 2D). The effects of NAC on Cd-induced inhibition of placental cell proliferation were then analyzed. Of interest, NAC obviously attenuated Cd-induced inhibition of cell proliferation in the placenta (Fig 2).

### NAC alleviates Cd–induced down-regulation of growth factors in mouse placenta

**Fig 3:**
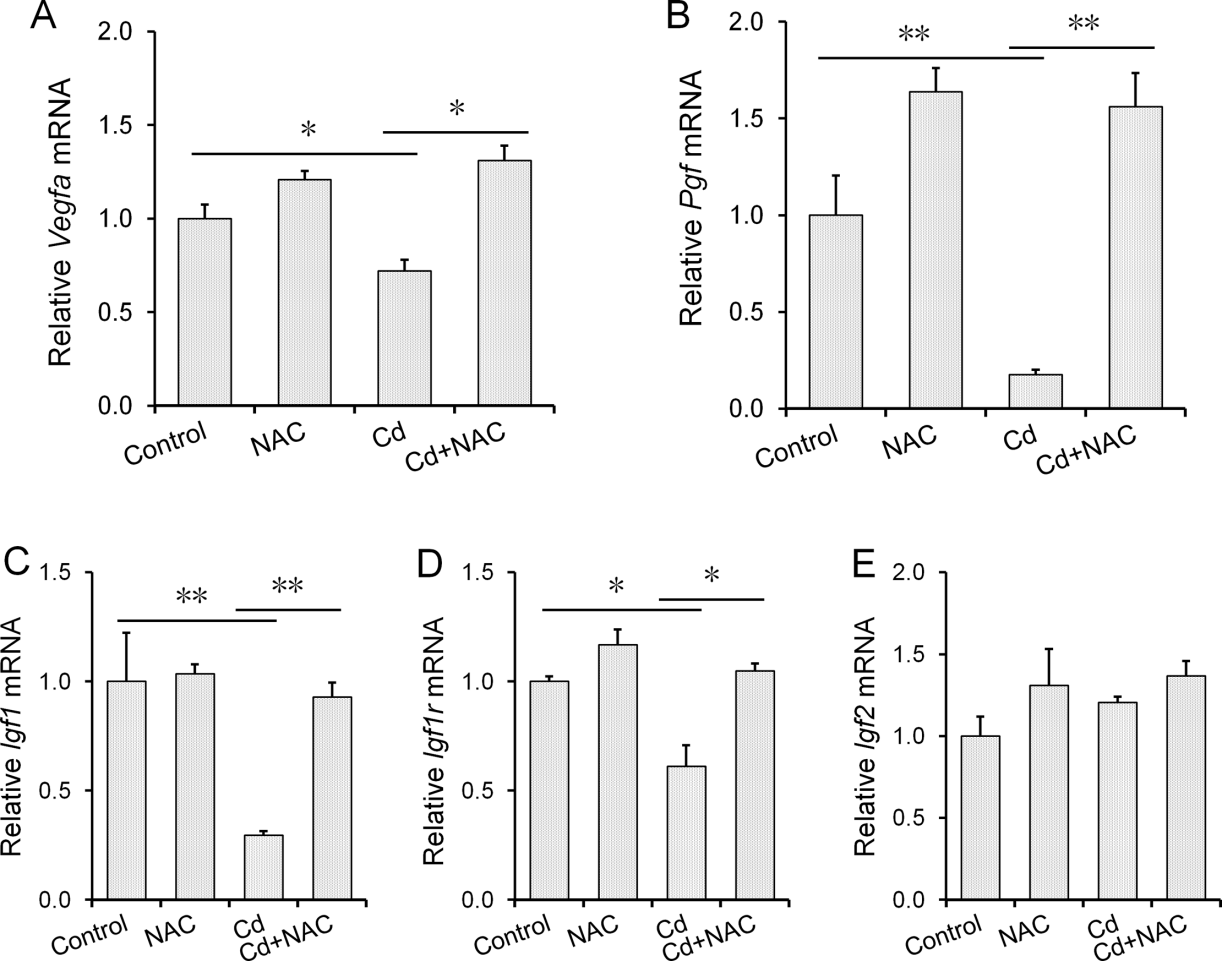
Effects of NAC on Cd-induced down-regulation of growth factors in mouse placenta. All pregnant mice except controls were i.p. injected with CdCl_2_ (1.0 mg/kg) daily from GD13 to GD15. In NAC+Cd group, pregnant mice were i.p. injected with NAC (1.0 g/kg) daily from GD12 to GD15, one dose (300 mg/kg) at 8 h before CdCl_2_ injection, one (400 mg/kg) at 30 min before CdCl_2_ injection, another (300 mg/kg) at 8 h after CdCl_2_ injection. Placentas were collected on GD 15. Placental *Vegf-α*, *Pgf*, *Igf1*, *Igf1r* and *Igf2* mRNAs were determined using real-time RT-PCR. (A) *Vegf-α*; (B) *Pgf*; (C) *Igf1*; (D) *Igf1r*; (E) *Igf2*. Data were expressed as means ± SEM. (N = 6). * *P*<0.05, **P<0.01.

The effects of maternal Cd exposure at late gestational stage on growth factors in mouse placenta are presented in Fig 3. As shown in Fig 3A, placental *Vegfα* mRNA level was reduced in Cd-exposed mice. In addition, *Pgf* expression was down-regulated in the placenta of mice exposed to Cd (Fig 3B). Although maternal Cd exposure at late gestational stage had little effect on placental *Igf2* expression (Fig 3E), placental *Igf1* and *Igf1r* mRNAs were reduced in Cd-exposed mice (Fig 3C and 3D). The effects of NAC on Cd-induced down-regulation of growth factors in placenta were analyzed. Interestingly, NAC significantly attenuated Cd-induced down-regulation of growth factors in mouse placenta (Fig 3A–3D).

### NAC alleviates Cd–induced down-regulation of placental nutrient transporters

**Fig 4:**
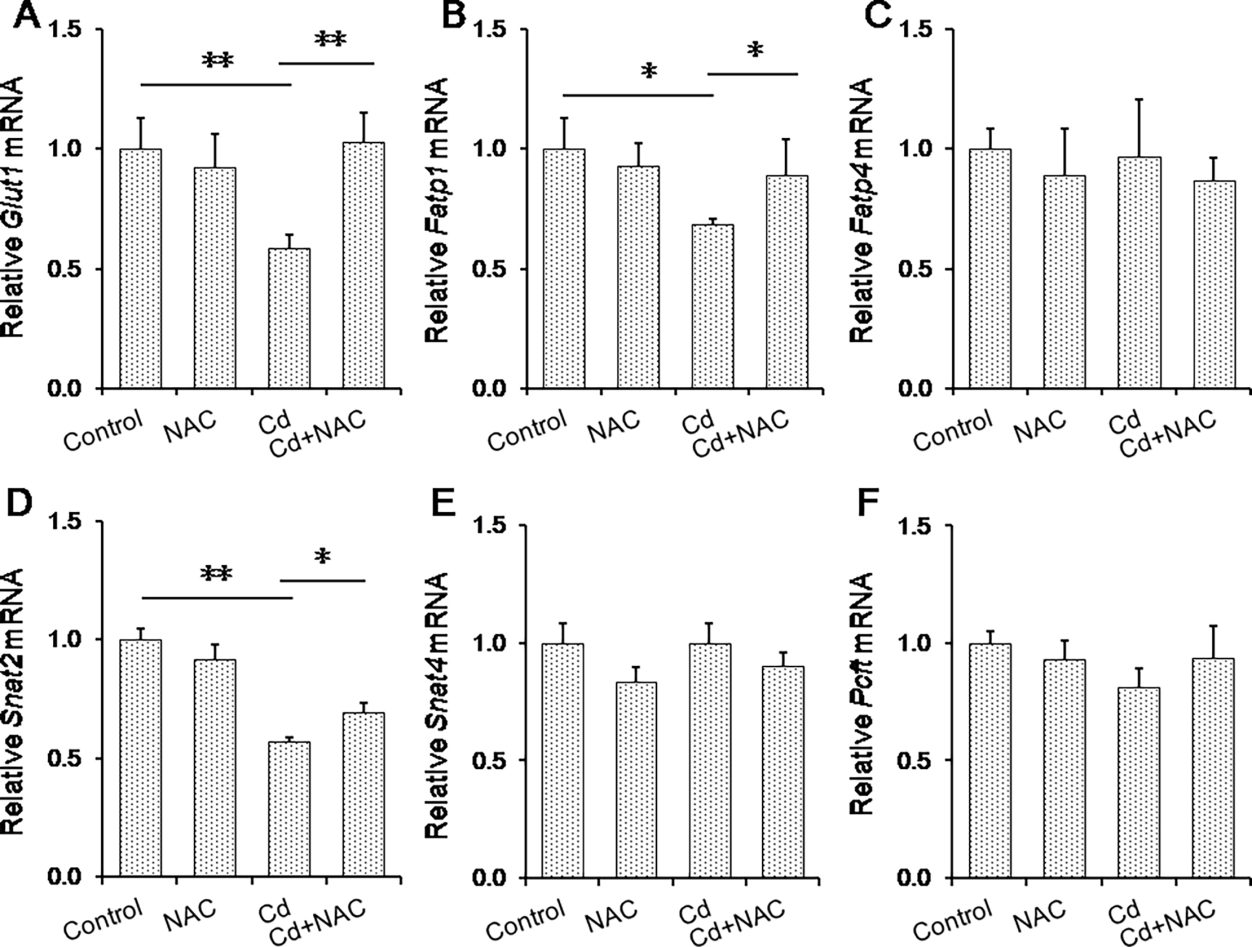
Effects of NAC on Cd-induced down-regulation of nutrient transport pumps in mouse placenta. All pregnant mice except controls were i.p. injected with CdCl_2_ (1.0 mg/kg) daily from GD13 to GD15. In NAC+Cd group, pregnant mice were i.p. injected with NAC (1.0 g/kg) daily from GD12 to GD15, one dose (300 mg/kg) at 8 h before CdCl_2_ injection, one (400 mg/kg) at 30 min before CdCl_2_ injection, another (300 mg/kg) at 8 h after CdCl_2_ injection. Placentas were collected on GD 15. Placental *Glut1*, *Fatp1*, *Fatp4*, *Snat2*, *Snat4* and *Pcft* mRNAs were determined using real-time RT-PCR. (A) *Glut1*; (B) *Fatp1*; (C) *Fatp4*; (D) *Snat2*; (E) *Snat4*. (F) *Pcft*. Data were expressed as means ± SEM. (N = 6). * *P*<0.05, **P<0.01.

The effects of maternal Cd exposure at late gestational stage on nutrient transport pumps in placenta are presented in Fig 4. As shown in Fig 4A, mRNA level of *Glut1*, a gene of a glucose transport pump, was significantly reduced in the placenta of mice exposed to Cd. Although maternal Cd exposure at late gestational stage had little effect on placental *Fatp4*, a fatty acid transport pump, mRNA levels of *Fatp1*, another fatty acid transport pump, was reduced in the placenta of Cd-exposed mice (Fig 4B and 4C). Maternal Cd exposure at late gestational stage reduced mRNA levels of *Snat2*, a gene of amino acid transport pump (Fig 4D). Maternal Cd exposure at late gestational stage had little effect on placental *Snat4*, another gene of amino acid transport pump, and *Pcft*, a gene of folic acid transport pump (Fig 4E and 4F). The effects of NAC on Cd-induced down-regulation of nutrient transport pumps in placenta were analyzed. Interestingly, NAC significantly attenuated Cd-induced down-regulation of nutrient transport pumps in mouse placenta (Fig 4A, 4B and 4D).

### NAC alleviates Cd-induced placental GSH depletion

**Fig 5:**
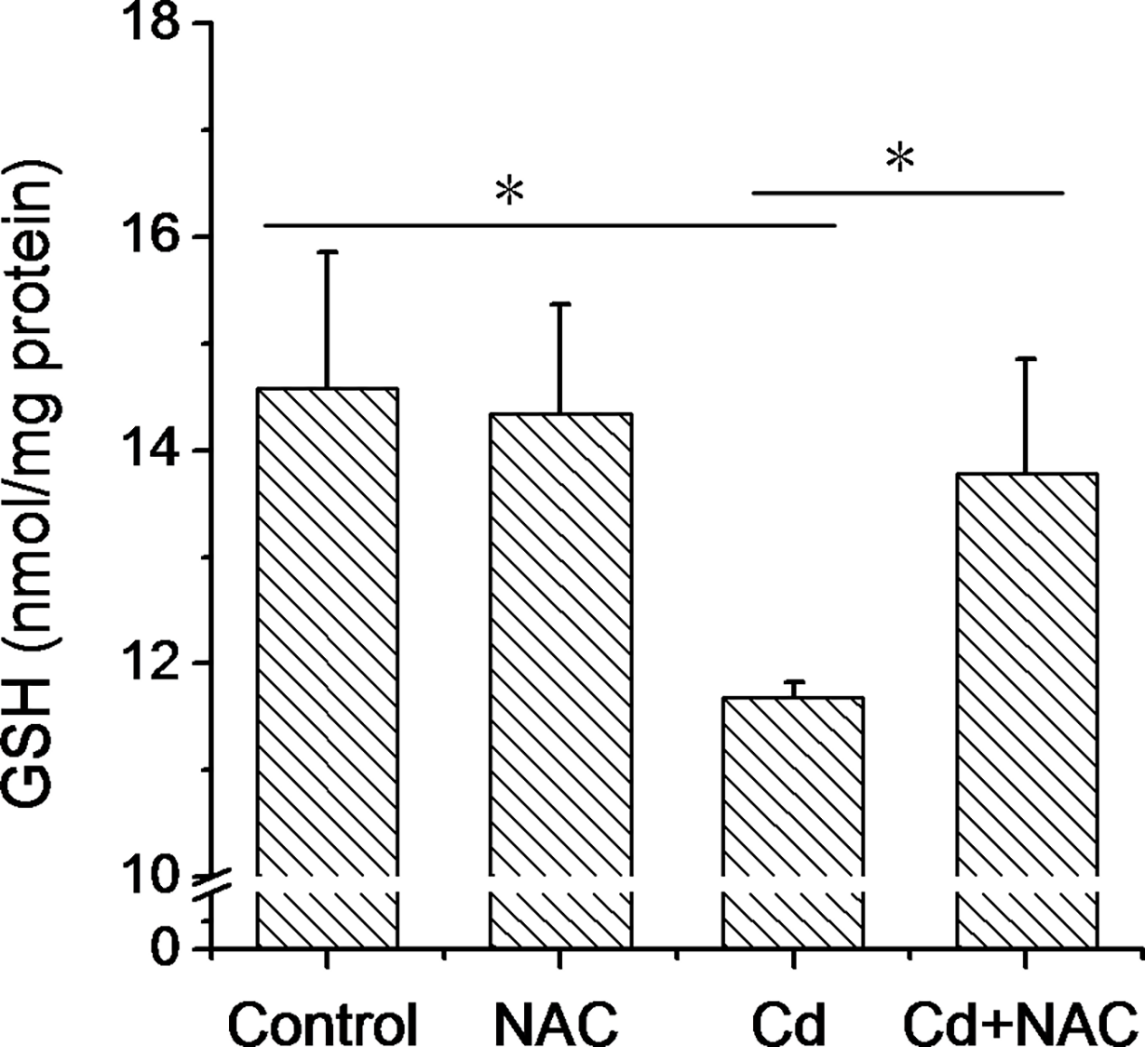
Effects of NAC on Cd-induced GSH depletion in mouse placenta. All pregnant mice except controls were i.p. injected with CdCl_2_ (1.0 mg/kg) daily from GD13 to GD17. In NAC+Cd group, pregnant mice were i.p. injected with NAC (1.0 g/kg) daily from GD12 to GD17, one dose (300 mg/kg) at 8 h before CdCl_2_ injection, one (400 mg/kg) at 30 min before CdCl_2_ injection, another (300 mg/kg) at 8 h after CdCl_2_ injection. Placentas were collected on GD 18. Placental GSH content was measured. All data were expressed as means ± SEM of six samples from six pregnant mice. * *P*<0.05.

The effects of NAC on placental GSH depletion were analyzed in placentas of mice exposed to Cd. As shown in Fig 5, placental GSH content was significantly reduced in placentas of mice exposed to Cd. NAC almost completely inhibited Cd-evoked placental GSH depletion (Fig 5).

### NAC attenuates Cd-induced placental ER stress

**Fig 6:**
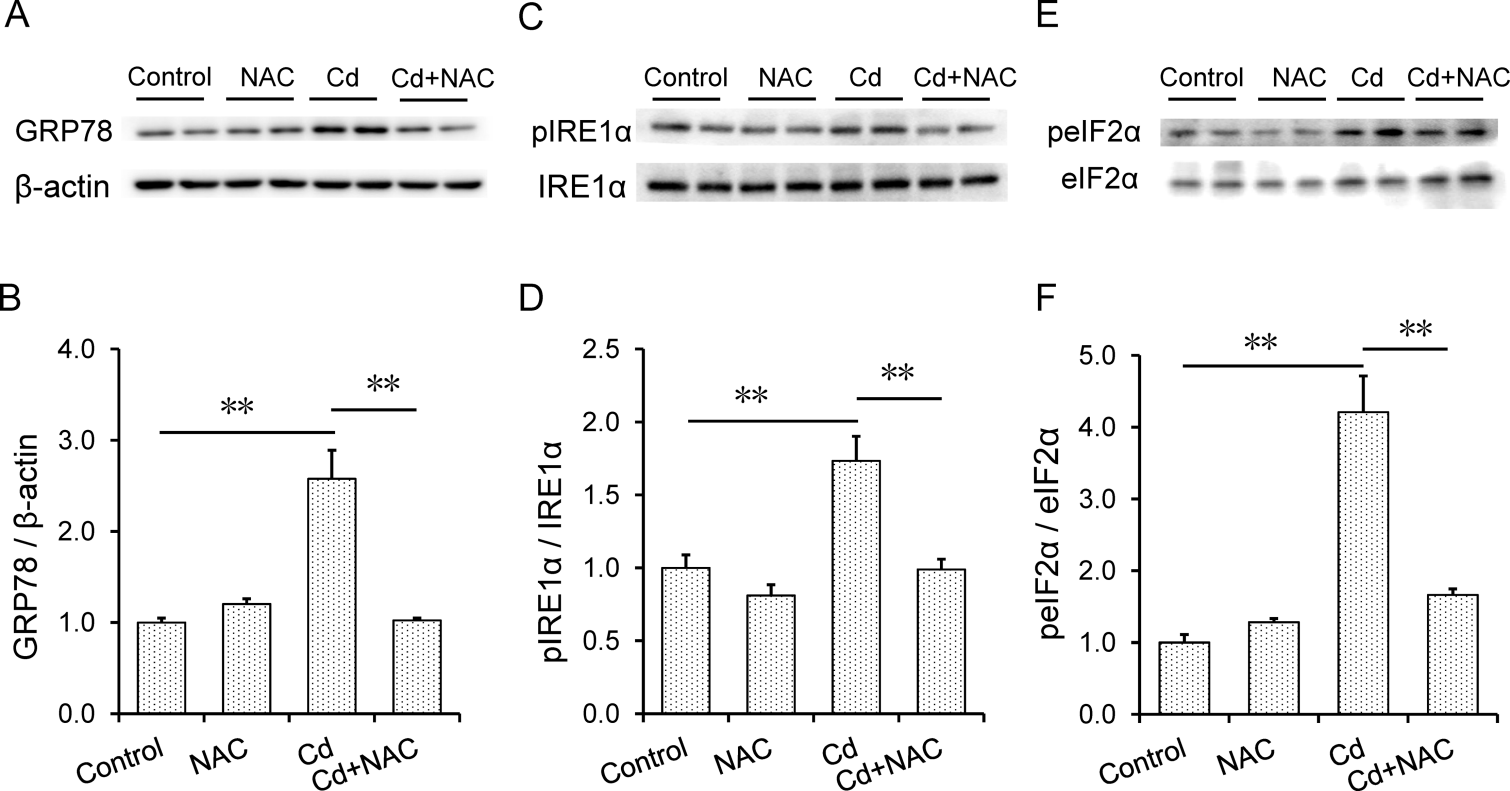
Effects of NAC on Cd-induced placental ER stress. All pregnant mice except controls were i.p. injected with CdCl_2_ (1.0 mg/kg) daily from GD13 to GD17. In NAC+Cd group, pregnant mice were i.p. injected with NAC (1.0 g/kg) daily from GD12 to GD17, one dose (300 mg/kg) at 8 h before CdCl_2_ injection, one (400 mg/kg) at 30 min before CdCl_2_ injection, another (300 mg/kg) at 8 h after CdCl_2_ injection. Placentas were collected on GD 18. Placental GRP78, pIRE1α and peIF2α were measured using immunoblots. (A) A representative gel for (A) GRP78, (C) pIRE1α and (E) peIF2α was shown. Please see the original bands for GRP78 and beta-actin in S1 and S2 Figs. All experiments were repeated for three times. (B, D, and F) All data were expressed as means ± SEM of six samples from six pregnant mice. **P<0.01.

The effects of maternal Cd exposure at late gestational stage on placental ER stress were analyzed. As expected, placental GRP78 protein was up-regulated in Cd-exposed mice (Fig 6A and 6B). Moreover, the level of phosphorylated IRE1α in placenta was elevated in Cd-exposed mice (Fig 6C and 6D). The level of phosphorylated eIF2α, a downstream target of the PERK pathway, was increased in placenta of mice exposed to Cd. (Fig 6E and 6F). Interestingly, NAC attenuated Cd-induced up-regulation of placental GRP78 (Fig 6A and 6B). In addition, NAC obviously inhibited Cd-induced phosphorylation of IRE1α and eIF2α in mouse placenta (Fig 6C–6F).

## Discussion

Our previous study mainly investigated the effects of maternal Cd exposure at the second trimester on fetal teratogenesis [8]. The previous results showed that maternal exposure to cadmium (4.5 mg/kg, i.p.) on gestational day 9 induced forelimb ectrodactyly in fetal mice, caused oxidative stress and endoplasmic reticulum stress in mouse placenta [8]. In the current study, we further explored the effects of maternal Cd exposure at the third trimester on fetal growth and development in mice. We found that maternal exposure to cadmium (1 mg/kg, i.p.) daily from gestation day 13 to 17 resulted in fetal growth restriction, induced oxidative stress and endoplasmic reticulum stress in mouse placenta. Further results showed that N-acetylcysteine (NAC) alleviates cadmium-induced placental endoplasmic reticulum stress and fetal growth restriction in mice.

An early study found that NAC prevented Cd-induced nephrotoxicity in rats [28]. A recent report showed that NAC protected mice from Cd-induced germ cell apoptosis in mouse testis [29]. In addition, NAC inhibited Cd-induced mitophagy in mouse brain [30]. In the present study, we investigated the effects of NAC on Cd-induced fetal growth restriction in mice. As expected, fetal weight and crown-rump length were significantly reduced in Cd-exposed mice. By contrast, FGR rate per litter was elevated in Cd-exposed mice. Of interest, NAC alleviated Cd-induced reduction of fetal weight and crown-rump length. These results suggest that NAC protects mice from Cd-induced fetal growth restriction.

The placenta is important for sustaining fetal growth and intrauterine development. The labyrinth is the site of oxygen and nutrient exchange between the mother and the fetus [31]. Increasing evidence demonstrates that placental insufficiency is major reason of fetal growth restriction [32–34]. Indeed, the present study showed that the weight of placenta in Cd-exposed mice was lower than in controls. Moreover, the percentage of Ki67-positive cells, a marker of cell proliferation, was decreased in the placenta of mice exposed to Cd. In addition, the internal space of maternal and fetal blood vessels in the labyrinth layer was reduced in the placenta of mice exposed to Cd, indicating that maternal Cd exposure induces placental insufficiency. To investigate whether NAC alleviates Cd-induced placental insufficiency, the present study analyzed the effects of NAC on cell proliferation and the internal space of vessels in mouse placenta. Of interest, NAC obviously alleviated Cd-induced impairment of maternal and fetal blood vessels in the labyrinth layer. In addition, NAC counteracted Cd-induced suppression of cell proliferation in the labyrinth layer. These results suggest that NAC might alleviate fetal growth through inhibiting Cd-induced placental insufficiency.

Increasing evidence demonstrated that ER stress was associated with the impaired placental development and fetal growth restriction [18–21]. A report from our laboratory showed that maternal Cd exposure on GD9 evoked placental ER stress [8]. Moreover, Cd-induced placental ER stress was associated with an increased incidence of forelimb ectrodactyly in fetuses [8]. The present study investigated the effects of maternal Cd exposure during late gestational stage on placental ER stress. We showed that placental GRP78 was up-regulated in mice exposed to Cd daily from GD13 to GD17. Moreover, the level of placental pIRE1α was elevated in mice exposed to Cd. In addition, the level of peIF2α, a downstream molecule of PERK signaling, was increased in the placenta of Cd-exposed mice. Of interest, NAC attenuated Cd-induced placental GRP78 up-regulation. In addition, NAC inhibited Cd-induced placental IRE1α and eIF2α phosphorylation. These results suggest that NAC might protect against Cd-induced fetal growth restriction, at least partially, through inhibiting ER stress in mouse placenta.

The mechanism through which ER stress mediates Cd-induced placental insufficiency and fetal growth restriction remains obscure. A recent study from our laboratory indicated that disturbance of placental folate transport contributed, at least partially, to Cd-induced neural tube defects and fetal growth restriction [9]. On the other hand, a recent study showed that mRNA levels of placental glucose transporter 1 (*Glut1*), a major transporter for glucose, placental growth factor (*Pgf*) and vascular endothelial growth factor receptor-1 (*Vegfr-1*), two vascular-related genes, were down-regulated under tunicamycin-evoked ER stress [21]. Further analysis found that placental ER stress negatively regulates transcription of placental growth factor via ATF4 and ATF6β [22]. In the present study, we investigated the effects of maternal Cd exposure on growth factors and nutrient transport pumps in mouse placenta. Our results showed that mRNA levels of placental *Vegfα*, *Pgf*, *Igf1* and *Igf1r*, several key genes of growth factors, were reduced in mice exposed to Cd daily from GD13 to GD17. In addition, placental *Glut1*, *Fatp1* and *Snat2*, several genes of nutrient transport pumps, were down-regulated in Cd-exposed mice. Of interest, NAC significantly attenuated Cd-induced down-regulation of growth factors and nutrient transport pumps in mouse placenta. These results suggest that maternal Cd exposure at late gestational stage induces placental insufficiency partially through ROS-mediated ER stress. Therefore, we guess that NAC alleviates Cd-induced fetal growth restriction, at least partially, through attenuating ER stress-mediated placental insufficiency. Additional experiments need to determine the protein expression of the placental growth factors and nutrient transporters in future studies.

The inhibitive effect of NAC on Cd-induced placental ER stress has preventive and therapeutic implications. An early study found that NAC reduced Cd-induced mesangial cell autophagy through inhibiting ROS-activated GSK-3β signaling [35]. Two recent studies showed that NAC protected against Cd-induced neuronal cell death through abrogating ROS-dependent activation of mTOR signaling [36, 37]. According to a recent report, NAC mediated Cd-induced mitophagy in mouse brain through blocking ROS-mediated activation of of PINK1/Parkin pathway [30]. Indeed, a report from our laboratory showed that NAC protected against Cd-induced germ cell apoptosis by inhibiting testicular ER stress [29]. The present study showed that NAC alleviated Cd-induced fetal growth restriction, at least partially, through inhibiting ER stress in mouse placenta. Our results suggest that NAC may be exploited for prevention and treatment of Cd-induced fetal growth restriction.

In summary, the present study investigated the effects of NAC on Cd-induced placental ER stress and fetal growth restriction in mice. Our results showed that NAC attenuated Cd-induced placental ER stress. We found that NAC prevented Cd-induced impairment of placental development. Importantly, NAC alleviated Cd-induced fetal growth restriction. We demonstrate that NAC may be exploited for prevention and treatment of Cd-induced placental insufficiency and fetal growth restriction.

## Supporting information

S1 Fig The original blot of GRP78 from Fig 6A. The order of samples are Control1, Control2, NAC1, NAC2, Cd1, Cd2, Cd+NAC1 and Cd+NAC2, respectively.
(TIF)

S2 Fig The original blot of beta-actin from Fig 6A. The order of samples are Control1, Control2, NAC1, NAC2, Cd1, Cd2, Cd+NAC1 and Cd+NAC2, respectively.
(TIF)

## Abbreviations

Cd: Cadmium
FGR: fetal growth restriction
ER stress: endoplasmic reticulum stress
NAC: N-acetylcysteine
GD: gestational day
IRE1α: inositol requiring ER-to-nucleus signal kinase
peIF2α: phosphor-eukaryotic translation initiation factor 2α
GSH: glutathione
pgf: placental growth factor

## References

[1] Hong J, Wang Y, McDermott S, Cai B, Aelion CM, Lead J. The use of a physiologically-based extraction test to assess relationships between bioaccessible metals in urban soil and neurodevelopmental conditions in children. Environ Pollut. 2016; 212: 9–17. doi: 10.1016/j.envpol.2016.01.001 26840511 10.1016/j.envpol.2016.01.001

[2] Wang H, Liu L, Hu YF, Hao JH, Chen YH, Su PY, et al Maternal serum cadmium level during pregnancy and its association with small for gestational age infants: a population-based birth cohort study. Sci Rep. 2016; 6: 22631 doi: 10.1038/srep22631 26934860 10.1038/srep22631 PMC4776171

[3] Wang H, Liu L, Hu YF, Hao JH, Chen YH, Su PY, et al Association of maternal serum cadmium level during pregnancy with risk of preterm birth in a Chinese population, Environ Pollut. 2016; 216: 851–857. doi: 10.1016/j.envpol.2016.06.058 27381872 10.1016/j.envpol.2016.06.058

[4] Yang J, Huo W, Zhang B, Zheng T, Li Y, Pan X, et al Maternal urinary cadmium concentrations in relation to preterm birth in the Healthy Baby Cohort Study in China. Environ Int. 2016; 94: 300–306. doi: 10.1016/j.envint.2016.06.003 27289180 10.1016/j.envint.2016.06.003

[5] Hovland DN Jr, Machado AF, Scott WJ Jr, Collins MD. Differential sensitivity of the SWV and C57BL/6 mouse strains to the teratogenic action of single administrations of cadmium given throughout the period of anterior neuropore closure. Teratology. 1999;60: 13–21. doi: 10.1002/(SICI)1096-9926(199907)60:1<13::AID-TERA6>3.0.CO;2-B 10413334 10.1002/(SICI)1096-9926(199907)60:1<13::AID-TERA6>3.0.CO;2-B

[6] Paniagua-Castro N, Escalona-Cardoso G, Chamorro-Cevallos G. Glycine reduces cadmium-induced teratogenic damage in mice. Reprod Toxicol. 2007; 23: 92–97. doi: 10.1016/j.reprotox.2006.08.011 17034988 10.1016/j.reprotox.2006.08.011

[7] Robinson JF, Yu X, Hong S, Griffith WC, Beyer R, Kim E, Faustman EM. Cadmium-induced differential toxicogenomic response in resistant and sensitive mouse strains undergoing neurulation. Toxicol Sci. 2009; 107: 206–19. doi: 10.1093/toxsci/kfn221 18974090 10.1093/toxsci/kfn221 PMC2768398

[8] Wang Z, Wang H, Xu ZM, Ji YL, Chen YH, Zhang ZH, et al Cadmium-induced teratogenicity: association with ROS-mediated endoplasmic reticulum stress in placenta. Toxicol Appl Pharmacol. 2012; 259: 236–247. doi: 10.1016/j.taap.2012.01.001 22252055 10.1016/j.taap.2012.01.001

[9] Zhang GB, Wang H, Hu J, Guo MY, Wang Y, Zhou Y, et al Cadmium-induced neural tube defects and fetal growth restriction: Association with disturbance of placental folate transport. Toxicol Appl Pharmacol. 2016; 306: 79–85. doi: 10.1016/j.taap.2016.07.007 27417525 10.1016/j.taap.2016.07.007

[10] Ahokas RA, Dilts PV Jr, LaHaye EB. Cadmium-induced fetal growth retardation: protective effect of excess dietary zinc. Am J Obstet Gynecol. 1980; 136: 216–221. 7352502 10.1016/0002-9378(80)90599-2

[11] Ji YL, Wang H, Liu P, Zhao XF, Zhang Y, Wang Q, et al Effects of maternal cadmium exposure during late pregnant period on testicular steroidogenesis in male offspring. Toxicol Lett. 2011; 205: 69–78. doi: 10.1016/j.toxlet.2011.05.233 21605642 10.1016/j.toxlet.2011.05.233

[12] Selvaratnam J, Guan H, Koropatnick J, Yang K. Metallothionein-I- and -II-deficient mice display increased susceptibility to cadmium-induced fetal growth restriction. Am J Physiol Endocrinol Metab. 2013; 305: E727–735. doi: 10.1152/ajpendo.00157.2013 23880315 10.1152/ajpendo.00157.2013

[13] Zhang S, Regnault TR, Barker PL, Botting KJ, McMillen IC, McMillan CM, et al Placental adaptations in growth restriction. Nutrients. 2015;7: 360–389. doi: 10.3390/nu7010360 25580812 10.3390/nu7010360 PMC4303845

[14] Wang M, Kaufman RJ. Protein misfolding in the endoplasmic reticulum as a conduit to human disease. Nature. 2016; 529: 326–335. doi: 10.1038/nature17041 26791723 10.1038/nature17041

[15] Ji YL, Wang H, Zhao XF, Wang Q, Zhang C, Zhang Y, et al Crosstalk between endoplasmic reticulum stress and mitochondrial pathway mediates cadmium-induced germ cell apoptosis in testes. Toxicol Sci. 2011;124: 446–459. doi: 10.1093/toxsci/kfr232 21908765 10.1093/toxsci/kfr232

[16] Luo B, Lin Y, Jiang S, Huang L, Yao H, Zhuang Q, et al Endoplasmic reticulum stress eIF2α-ATF4 pathway-mediated cyclooxygenase-2 induction regulates cadmium-induced autophagy in kidney. Cell Death Dis. 2016;7: e2251 doi: 10.1038/cddis.2016.78 27253415 10.1038/cddis.2016.78 PMC5143407

[17] Iwawaki T, Akai R, Yamanaka S, Kohno K. Function of IRE1 alpha in the placenta is essential for placental development and embryonic viability. Proc Natl Acad Sci U S A. 2009;106: 16657–16662. doi: 10.1073/pnas.0903775106 19805353 10.1073/pnas.0903775106 PMC2757843

[18] Lian IA, Loset M, Mundal SB, Fenstad MH, Johnson MP, Eide IP, et al Increased endoplasmic reticulum stress in decidual tissue from pregnancies complicated by fetal growth restriction with and without pre-eclampsia. Placenta. 2011; 32: 823–829. doi: 10.1016/j.placenta.2011.08.005 21907405 10.1016/j.placenta.2011.08.005 PMC3210381

[19] Yung HW, Calabrese S, Hynx D, Hemmings BA, Cetin I, Charnock-Jones DS, et al Evidence of placental translation inhibition and endoplasmic reticulum stress in the etiology of human intrauterine growth restriction. Am J Pathol. 2008; 173: 451–462. doi: 10.2353/ajpath.2008.071193 18583310 10.2353/ajpath.2008.071193 PMC2475782

[20] Yung HW, Hemberger M, Watson ED, Senner CE, Jones CP, Kaufman RJ, et al Endoplasmic reticulum stress disrupts placental morphogenesis: implications for human intrauterine growth restriction. J Pathol. 2012; 228: 554–564. doi: 10.1002/path.4068 22733590 10.1002/path.4068 PMC3532660

[21] Kawakami T, Yoshimi M, Kadota Y, Inoue M, Sato M, Suzuki S, Prolonged endoplasmic reticulum stress alters placental morphology and causes low birth weight. Toxicol. Appl Pharmacol. 2014; 275: 134–144. doi: 10.1016/j.taap.2013.12.008 24370435 10.1016/j.taap.2013.12.008

[22] Mizuuchi M, Cindrova-Davies T, Olovsson M, Charnock-Jones DS, Burton GJ, Yung HW. Placental endoplasmic reticulum stress negatively regulates transcription of placental growth factor via ATF4 and ATF6β: implications for the pathophysiology of human pregnancy complications. J Pathol. 2016; 238: 550–561. doi: 10.1002/path.4678 26648175 10.1002/path.4678 PMC4784173

[23] Rushworth GF, Megson IL. Existing and potential therapeutic uses for N-acetylcysteine: the need for conversion to intracellular glutathione for antioxidant benefits. Pharmacol Ther. 2014; 141: 150–159. doi: 10.1016/j.pharmthera.2013.09.006 24080471 10.1016/j.pharmthera.2013.09.006

[24] Deng X, Xia Y, Hu W, Zhang H, Shen Z, Cadmium-induced oxidative damage and protective effects of N-acetyl-L-cysteine against cadmium toxicity in Solanum nigrum L. J Hazard Mater. 2010; 180: 722–729. doi: 10.1016/j.jhazmat.2010.04.099 20488618 10.1016/j.jhazmat.2010.04.099

[25] Balansky R, Agostini FD, Ganchev G, Izzotti A, Marco BD, Lubet RA, et al Influence of FHIT on benzo [a] pyrene-induced tumors and alopecia in mice: chemoprevention by budesonide and N-acetylcysteine. Proc Natl Acad Sci U S A. 2006; 103: 7823–7828. doi: 10.1073/pnas.0601412103 16672365 10.1073/pnas.0601412103 PMC1472529

[26] Chen YH, Hu XG, Zhou Y, Yu Z, Fu L, Zhang GB, et al Obeticholic acid orotects against lipopolysaccharide-induced fetal death and intrauterine growth restriction through its anti-inflammatory activity. J Immunol. 2016; 197: 4762–4770. doi: 10.4049/jimmunol.1601331 27821667 10.4049/jimmunol.1601331

[27] Griffith OW. Determination of glutathione and glutathione disulfide using glutathione reductase and 2-vinylpyridine. Anal Biochem. 1980; 106: 207–212. 7416462 10.1016/0003-2697(80)90139-6

[28] Shaikh ZA, Zaman K, Tang W, Vu T. Treatment of chronic cadmium nephrotoxicity by N-acetyl cysteine. Toxicol Lett. 1999; 104: 137–142. 10048759 10.1016/s0378-4274(98)00358-0

[29] Ji YL, Wang H, Zhang C, Zhang Y, Zhao M, Chen YH, et al N-acetylcysteine protects against cadmium-induced germ cell apoptosis by inhibiting endoplasmic reticulum stress in testes. Asian J Androl. 2013; 15: 290–296. doi: 10.1038/aja.2012.129 23353715 10.1038/aja.2012.129 PMC3739146

[30] Wei X, Qi Y, Zhang X, Gu X, Cai H, Yang J, et al ROS act as an upstream signal to mediate cadmium-induced mitophagy in mouse brain. Neurotoxicology. 2015; 46: 19–24. doi: 10.1016/j.neuro.2014.11.007 25464205 10.1016/j.neuro.2014.11.007

[31] Dimasuay KG, Boeuf P, Powell TL, Jansson T. Placental Responses to Changes in the Maternal Environment Determine Fetal Growth. Front Physiol. 2016;7: 12 doi: 10.3389/fphys.2016.00012 26858656 10.3389/fphys.2016.00012 PMC4731498

[32] Conroy AL, Silver KL, Zhong K, Rennie M, Ward P, Sarma JV, et al Complement activation and the resulting placental vascular insufficiency drives fetal growth restriction associated with placental malaria. Cell Host Microbe. 2013; 13: 215–226. doi: 10.1016/j.chom.2013.01.010 23414761 10.1016/j.chom.2013.01.010

[33] Walker CK, Krakowiak P, Baker A, Hansen RL, Ozonoff S, et al Preeclampsia, placental insufficiency, and autism spectrum disorder or developmental delay. JAMA Pediatr. 2015;169: 154–162. doi: 10.1001/jamapediatrics.2014.2645 25485869 10.1001/jamapediatrics.2014.2645 PMC4416484

[34] Williams CJ, Chu A, Jefferson WN, Casero D, Sudhakar D, Khurana N, et al Epithelial membrane protein 2 (EMP2) deficiency alters placental angiogenesis, mimicking features of human placental insufficiency. J Pathol. 2017; 242: 246–259. doi: 10.1002/path.4893 28295343 10.1002/path.4893 PMC5444952

[35] Wang SH, Shih YL, Kuo TC, Ko WC, Shih CM. Cadmium toxicity toward autophagy through ROS-activated GSK-3beta in mesangial cells. Toxicol Sci. 2009, 108: 124–131. doi: 10.1093/toxsci/kfn266 19126599 10.1093/toxsci/kfn266

[36] Chen L, Xu B, Liu L, Luo Y, Zhou H, Chen W, et al Cadmium induction of reactive oxygen species activates the mTOR pathway, leading to neuronal cell death. Free Radic Biol Med. 2011; 50: 624–632. doi: 10.1016/j.freeradbiomed.2010.12.032 21195169 10.1016/j.freeradbiomed.2010.12.032 PMC3032035

[37] Chen S, Ren Q, Zhang J, Ye Y, Zhang Z, Xu Y, et al N-acetyl-L-cysteine protects against cadmium-induced neuronal apoptosis by inhibiting ROS-dependent activation of Akt/mTOR pathway in mouse brain. Neuropathol Appl Neurobiol. 2014; 40: 759–777. doi: 10.1111/nan.12103 24299490 10.1111/nan.12103 PMC4043941

