# Supplementary figures and images for: N-acetylcysteine alleviates cadmium-induced placental endoplasmic reticulum stress and fetal growth restriction in mice
Source: PLoS One. 2018 Jan 26;13(1):e0191667. doi: 10.1371/journal.pone.0191667 (PMC5786300; doi:10.1371/journal.pone.0191667)

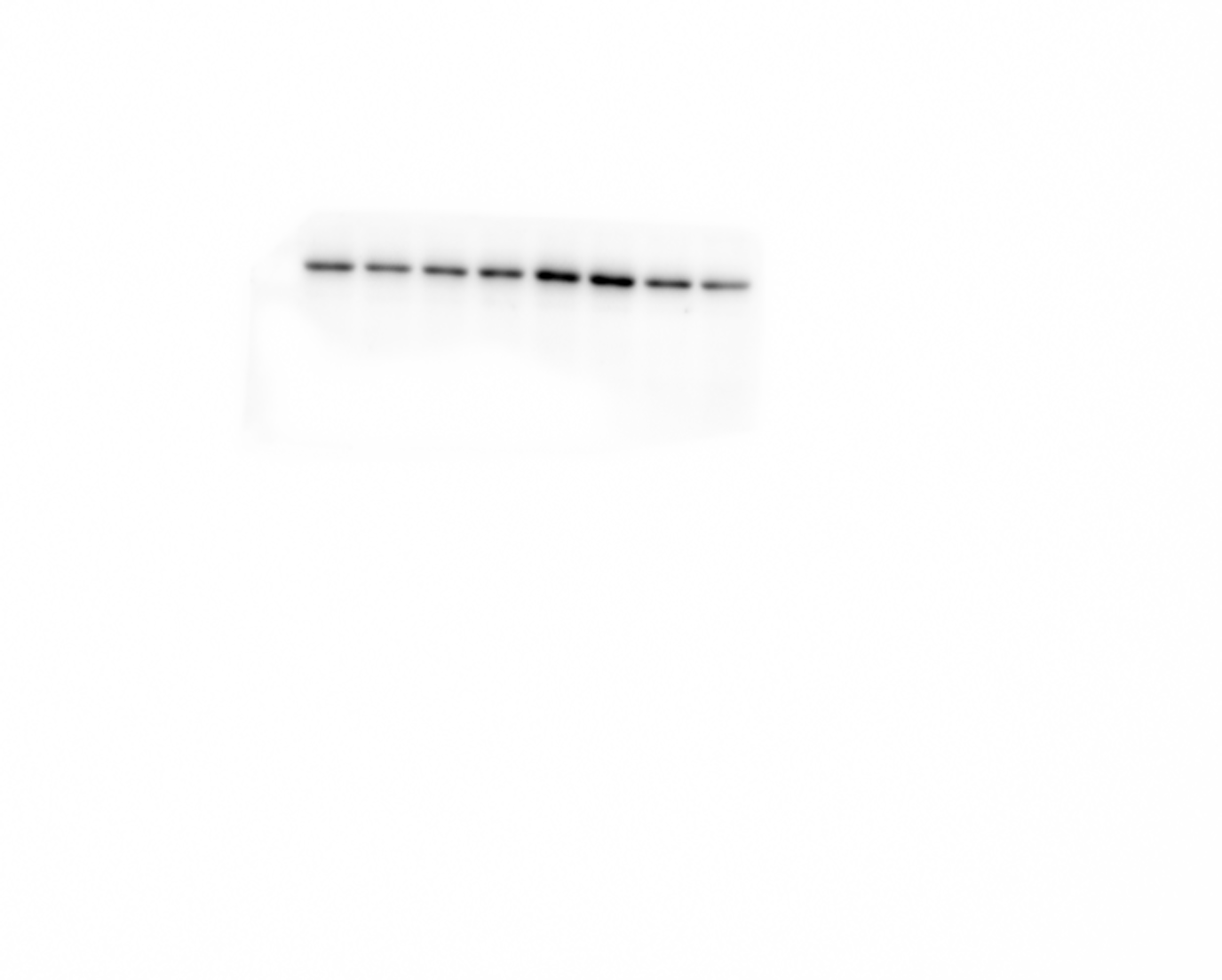

Supplement: S1 Fig — The order of samples are Control1, Control2, NAC1, NAC2, Cd1, Cd2, Cd+NAC1 and Cd+NAC2, respectively. (TIF) [file pone.0191667.s001.tif]

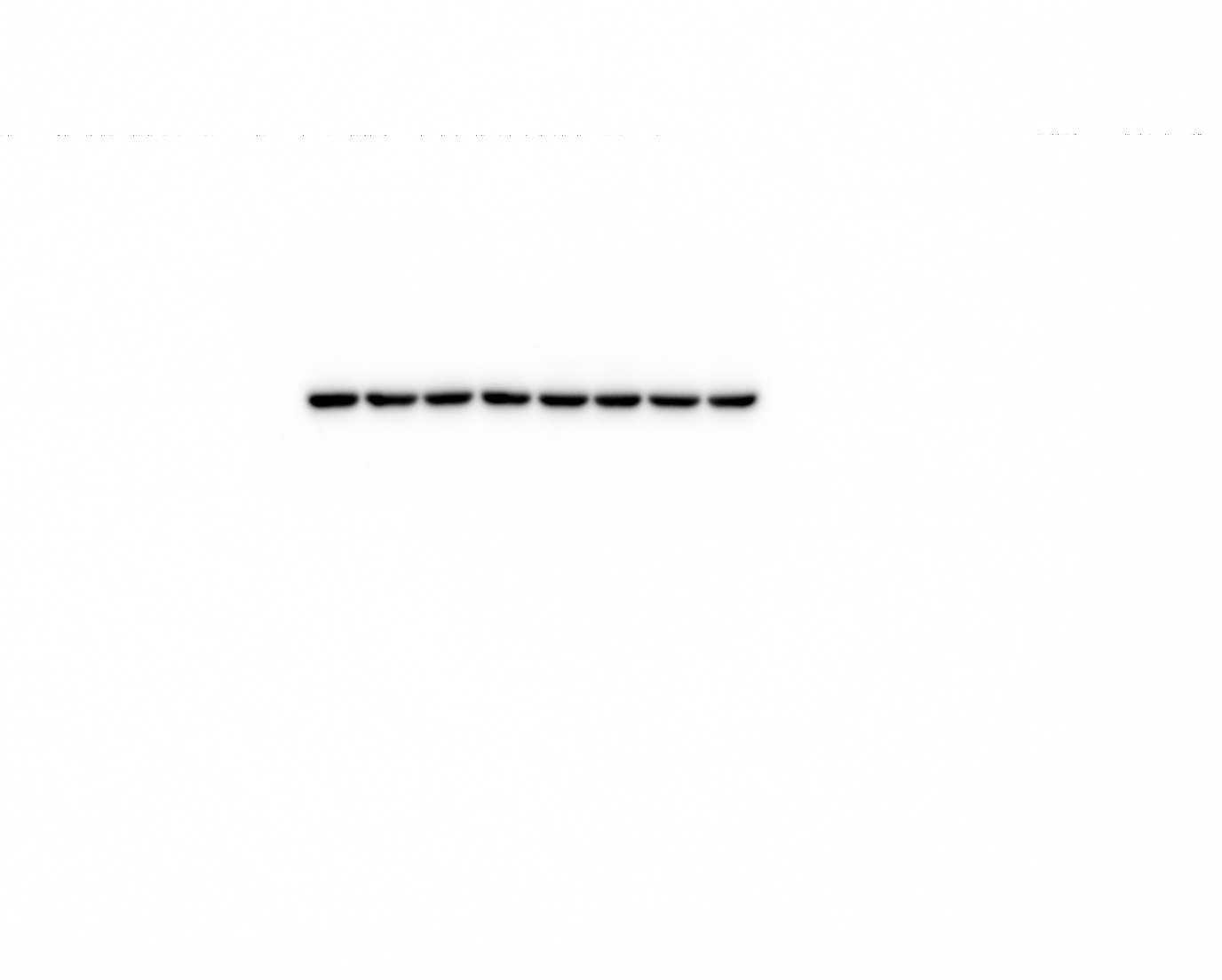

Supplement: S2 Fig — The order of samples are Control1, Control2, NAC1, NAC2, Cd1, Cd2, Cd+NAC1 and Cd+NAC2, respectively. (TIF) [file pone.0191667.s002.tif]
